# 

**Published:** 1890-09

**Authors:** 


### PENNSYLVANIA STATE VETERINARY MEDICAL ASSOCIATION.

THE semi-annual meeting of the Pennsylvania State Veterinary Medical Association will be held at City Hotel, Lancaster city, Pa., September 2d, 1890, at 10 o'clock A.M. A large meeting is looked for, as some forty new names will be presented for membership and important matters will be discussed.

DR. ROBERT GLADFELTFR, *Secretary*.

### BOOKS AND PAMPHLETS RECEIVED.

Contributions to the Comparative Osteology of Arctic and Sub-Arctic Water Birds. Part VII. By R. W. Shufeldt, M.D., C.M.Z.S. Reprinted from *Jour. Anatomy and Physiology*, Vol. XXIV.

Varicocele. By Thomas W. Kay, M.D., Scranton, Pa.

Thiermedizinische Vortrage. Edited by Dr. George Schneidemuhl. Bd. 11, Heft 1. Leipzig.

Bollettino Scientifico. Anno XII, No. 2. Pavia.

The Journal of the Cicinnati Society of Natural History. Vol. XIII, No. 2.

Bulletin Mensuel Societé Linneenne du Nord de la France. Nos. 209–214. Amiens.

Bulletin de la Societé Zoologique de France. T. XV, No. 6 Paris.

Leopoldina. Amtliches organ der K. Leop. Carol Deutschen Akademie der Naturforscher. Edited by Dr. C. H. Knoblauch. Halle, 1889.

Annales et Bulletin de la Societé de Medecine de Gand.

Anales de la Sociedad Cientifica. Argentina. Buenos Ayres, 1890.

Annual Report of the New South Wales Zoological Society. Sydney, 1890.

Ottawa Naturalist.

